# Identification and validation of biomarkers associated with mitotic catastrophe in high-altitude hypoxia

**DOI:** 10.1186/s41065-026-00692-9

**Published:** 2026-05-21

**Authors:** Wenwen Zhao, Defang Liu, Ting Luo, Yang Wu

**Affiliations:** Traditional Chinese Medicine Department, The General Hospital of Western Theater Command PLA, Chengdu, China

**Keywords:** High-altitude hypoxia, Mitotic catastrophe, Biomarkers, Nomogram

## Abstract

**Background:**

High-altitude hypoxia (HAH) can cause adverse reactions, such as tinnitus and barotrauma, but the role of mitotic catastrophe (MC) in HAH remains unreported. This study investigated MC-associated biomarkers in HAH.

**Methods:**

HAH-related datasets and MC-related genes (MC-RGs) were retrieved from public databases. Differentially expressed genes (DEGs) between the HAH and control groups were screened, and overlapping genes between DEGs and MC-RGs were defined as candidates. Biomarkers were identified using three machine learning methods, receiver operating characteristic curve analysis, and expression validation. A nomogram was constructed for diagnostic assessment. Functional enrichment, immune infiltration, regulatory network, and drug prediction analyses were performed, with biomarker expression verified by reverse transcription quantitative polymerase chain reaction (RT-qPCR).

**Results:**

*DIAPH1* and *MDM4* were confirmed as MC-associated biomarkers in HAH, and the nomogram displayed excellent diagnostic ability (area under the curve = 0.929). These biomarkers were coenriched in pathways such as oxidative phosphorylation. *DIAPH1* was positively correlated with the infiltration of resting NK cells, whereas *MDM4* was negatively correlated with that of M2 macrophages. A transcription factor–mRNA–miRNA network was established, with *USF2* and *GATA2* jointly targeting both biomarkers. Drugs such as rescinnamine targeted *MDM4*. RT-qPCR verified that *USF2* and *GATA2* were significantly downregulated in HAH (both *P* < 0.01), consistent with the bioinformatics results.

**Conclusions:**

*DIAPH1* and *MDM4* are valid MC-associated biomarkers for HAH. This study provides novel insights into HAH prevention and treatment.

**Supplementary Information:**

The online version contains supplementary material available at https://doi.org/10.1186/s41065-026-00692-9.

## Background

High-altitude hypoxia (HAH) describes the pathophysiological state in which individuals experience tissue and organ hypoxia because of decreased atmospheric pressure and oxygen partial pressure at elevations exceeding 2500 m [1]. HAH significantly affects the health of native high-altitude populations, migrants, travelers, and workers engaged in high-altitude operations [2]. This condition both increases the medical burden and potentially hinders economic development in high-altitude regions. The pathogenesis of HAH involves complex pathophysiological processes, with existing evidence demonstrating its close association with blood–brain barrier (BBB) disruption [3] and hypoxia-induced free radicals [4]. Prolonged or severe hypoxia triggers a cascade of damaging effects, including oxidative stress caused by excessive reactive oxygen species (ROS) production [5], mitochondrial dysfunction leading to insufficient ATP synthesis [6], endoplasmic reticulum stress and activation of the unfolded protein response [7], and chronic inflammatory states induced by pro-inflammatory cytokine release [8]. These pathological changes ultimately result in damage to vital organs such as the heart, brain, lungs, and reproductive system [9, 10]. Current clinical management primarily relies on oxygen therapy, rest, and symptomatic treatment, which can alleviate symptoms to some extent; however, specific interventions that halt disease progression are lacking. Therefore, identifying biomarkers for early warning and disease progression assessment is important for improving the diagnosis and treatment system for HAH.

Mitotic catastrophe (MC) is a form of cell death caused by abnormal mitotic processes when cells experience damage or stress, characterized by disorders of chromosome segregation and the formation of multinucleated cells, etc [11]. The occurrence of MC involves processes such as loss of control of cell cycle checkpoints, abnormal spindle assembly, and erroneous chromosome segregation [12]. Recent studies have found that severe hypoxic stress can interfere with the mitotic process through multiple pathways, such as disrupting the spindle assembly checkpoint, damaging centrioles, or interfering with DNA damage repair, ultimately inducing MC [13]. Although hypoxia can induce MC in diseases such as diabetes and tumors, research on this phenomenon in HAH remains insufficient [14]. Therefore, identifying and verifying specific biomarkers related to MC in HAH is of great significance, as it can provide a molecular basis for early warning, disease assessment, and targeted therapy.

This study employed bioinformatics analysis methods to identify differentially expressed genes (DEGs) associated with HAH. Potential candidate biomarkers are screened using machine learning algorithms, and their diagnostic efficacy was evaluated *via* receiver operating characteristic (ROC) curve analysis alongside expression validation. Subsequently, functional enrichment analysis was conducted to elucidate the involved biological processes and signaling pathways. Immune infiltration analysis was performed to probe the associations of the biomarkers with immune cell infiltration, and regulatory networks were constructed to decipher potential molecular mechanisms. Drug prediction analysis was conducted to identify potential therapeutic candidates, followed by validation in clinical samples to assess their clinical applicability. The ultimate goal was to provide a scientific foundation for the diagnosis, treatment, and prognosis evaluation of HAH.

## Methods

### Data collection

The HAH datasets (GSE46480 and GSE103940) were obtained from the GEO dataset. GSE46480 (platform: GPL570) included transcriptome data from 98 HAH and 98 control peripheral blood mononuclear cell samples. GSE103940 (platform: GPL11154) included transcriptome data from 11 HAH and 11 control blood samples. All samples were obtained from humans. Moreover, 1213 genes linked to MC-related genes (MC-RGs) were retrieved from MCDB (Additional file 1). The research data for this study were accessed on October 16, 2024.

### Differential expression analysis

Using the limma package (v 3.58.1) [15], we analyzed DEGs between HAH and control samples in GSE46480 (*P* < 0.05, |log_2_ fold change [FC]| > 0.5). Subsequently, the ggplot2 package (v 3.4.4) [16] and ComplexHeatmap package (v 2.18.0) [17] were employed to generate volcano plots and heatmaps, respectively, which were used to visualize these DEGs.

### Identification and enrichment analyses of candidate genes

DEGs and MC-RGs were intersected to identify candidate genes. The ggvenn package (v 1.4.9) was adopted to create the Venn diagram. These candidate genes (P.adj < 0.05) were then subjected to Gene Ontology (GO) and Kyoto Encyclopedia of Genes and Genomes (KEGG) enrichment analyses using the clusterProfiler program (v 4.10.0) [18]. The STRING database (https://string-db.org/) was applied to build a protein–protein interaction (PPI) network to probe the interplay of potential genes at the protein level. The interaction score was set to greater than 0.4, and the species was specified as “*Homo sapiens*.” The constructed PPI network was displayed using Cytoscape software (v 3.10.2) [19].

### Machine learning

Based on the candidate genes, the glmnet package (v 4.1.8) [20] was employed to conduct Least Absolute Shrinkage and Selection Operator (LASSO) regression analysis. When lambda reached its minimum value and the coefficients of genes were larger than 0, feature gene set 1 was identified. For LASSO regression, 10‑fold cross‑validation was applied. The caret package (v 6.0.94) [21] was used to conducted SVM-RFE, for which 10-fold cross-validation was also conducted. At the moment when the accuracy rate peaked, feature gene set 2 was identified. Boruta analysis was performed concurrently using the Boruta package (v 8.0.0) [22]. Feature gene set 3 was selected by ranking the importance of the genes. Subsequently, the genes resulting from the overlap of feature gene sets 1, 2, and 3 through the ggvenn package were regarded as the key genes.

### Identification of biomarkers

The pROC package (v 1.18.5) [23] was adopted to plot ROC curves in GSE46480 and GSE103940. As candidate biomarkers, the genes that met area under the curve (AUC) > 0.7 in both GSE46480 and GSE103940 were selected. AUC > 0.7 indicated that the gene had a good capacity to distinguish between HAH and control samples. Next, the rstatix package (v 0.7.2) was adopted to analyze the differences in potential biomarker expression between HAH and control samples in GSE46480 and GSE103940 using the Wilcoxon test (*P* < 0.05). Genes exhibiting stable expression trends and notable expression differences between HAH and control samples were deemed biomarkers in GSE46480 and GSE103940.

### Construction and evaluation of the nomogram

A nomogram was created in GSE46480 *via* the rms package (v 6.5.0) [24] to investigate the prediction power of biomarkers for the presence of HAH. The calibration curve was then plotted through the rms package to judge the nomogram model’s prediction accuracy. Additionally, the ROC curve was constructed using the pROC package. A nomogram model was considered to have good predictive accuracy when its AUC exceeded 0.7.

### Functional analyses of biomarkers

GSEA was performed to understand the biological functions and pathways in which that biomarkers potentially participate. First, according to the median expression of each biomarker, all GSE46480 samples were split into high- and low-expression groups. The limma package was then applied to perform differential expression analysis between these groups, and log_2_FC was computed. The genes were ranked according to log_2_FC from largest to smallest. Subsequently, GSEA was undertaken using the clusterProfiler package (v 4.10.0; |NES| > 1, P.adj < 0.05). The background gene set “c2.cp.kegg.v7.5.1.symbols.gmt” was obtained from MSigDB. The GseaVis package (v 0.1.0) and GSEABase package (v 1.66.0) were used to visualize the results. Next, to explore genes with similar functions to the biomarkers and their functions, the gene–gene interaction (GGI) network was created using the GeneMANIA database (https://genemania.org/).

### Immune infiltration analysis

This study applied the CIBERSORT algorithm to analyze the infiltration density of 22 immune cell types [25] in HAH and control samples from GSE46480, constructing overlay plots using the ggplot2 package (*P* > 0.05). Subsequently, the Wilcoxon test in rstatix was adopted to analyze differences in the infiltration level of these immune cells (*P* < 0.05). Box plots were generated using the ggpubr package (v 0.6.0) to visualize the results [26]. The Spearman correlation between differentially infiltrating immune cells and their related biomarkers (|cor| > 0.3, *P* < 0.05) was assessed using the psych package (v 2.4.3). Finally, correlation heatmaps were generated using the corrplot program (v 0.92) to visualize the findings [27]. To further validate the results of the CIBERSORT algorithm, we used the ssGSEA algorithm to calculate scores for the infiltration levels of 28 types of immune cells between the HAH and healthy control groups in the GSE46480 dataset, and differences in immune cell infiltration between these groups were compared using the Wilcoxon rank-sum test.

### Regulatory network analysis

Transcription factors (TFs) regulating the biomarkers were predicted using the Joint Annotation of Protein Affinity Regulating database within the NetworkAnalyst platform. miRNAs targeting biomarkers were predicted separately using the miRWalk, miRDB, and TargetScan databases. Then, the intersection of the miRNAs predicted by the three databases was used to construct the subsequent TF–mRNA–miRNA regulatory network.

### Drug prediction

The DGIdb database was employed to forecast drugs that target biomarkers (*P* < 0.05). Subsequently, the top 15 drugs based on the P-value were selected and ranked from smallest to largest, and a drug–mRNA network was constructed. After selecting one of these drugs for molecular docking, the drug’s 2D structure was first downloaded from PubChem (https://pubchem.ncbi.nlm.nih.gov/). Next, the protein crystal structure with the highest match to the biomarker was downloaded from the PDB database (https://www.rcsb.org/). Subsequently, molecular docking was performed using the cbdock2 website (http://183.56.231.194:8001/cb-dock2/index.php). To display this network, a Sankey diagram was created using the ggsankey package (v 0.0.99999). The Herb database (http://herb.ac.cn/) was searched for Chinese herbal medications that target the biomarkers. The Cytoscape package was applied to generate and illustrate a Chinese herbal medicine–mRNA network.

### Reverse transcription quantitative polymerase chain reaction (RT-qPCR)

To preliminarily investigate the mRNA expression of biomarkers in clinical samples, this study collected blood samples from patients with HAH (*n* = 5) and healthy controls (*n* = 5) from The General Hospital of Western Theater Command PLA for RT-qPCR. The samples were collected on August 3, 2025. Total RNA was extracted from each sample using TRIzol reagent and reverse-transcribed using HP All-in-one qRT Master Mix II RT203-Ver.1 reverse transcriptase. RT-qPCR was conducted using the 2× Universal Blue SYBR Green qPCR Master Mix kit, and expression differences between samples were analyzed using a *t*-test (*P* < 0.05). Gene expression was quantified using the 2^−ΔΔCt^ method. The primers used were prepared by Sangon Biotech (Shanghai) Co., Ltd (Additional file 2). Each biological sample was subjected to three technical replicates. Meanwhile, this study has obtained approval from the Ethics Committee of the General Hospital of Western Theater Command of the PLA (2024EC1-ky024). *GAPDH* was applied as the internal reference gene. The obtained data were statistically analyzed and presented using GraphPad Prism (v 10.0, GraphPad, Boston, MA, USA) [28].

### Statistical analysis

For statistical analysis, R (v. 4.3.2, R Foundation for Statistical Computing, Vienna, Austria) was utilized. The Wilcoxon test was adopted to examine between-group differences (*P* < 0.05). A *t*-test was employed in RT-qPCR studies to compare expression differences between the groups (*P* < 0.05).

## Results

### Screening of 84 candidate genes

Differential expression analysis identified 1006 DEGs between the HAH and control groups, including 986 upregulated and 20 downregulated genes. Using volcano plot analysis, the top 10 most strongly upregulated and downregulated DEGs were screened by descending |log_2_FC| (Fig. 1a), and their expression was visualized *via* a heatmap (Fig. 1b). The 1006 DEGs were intersected with 1213 MC-RGs, yielding 84 candidate genes (Fig. 1c).

**Fig. 1:**
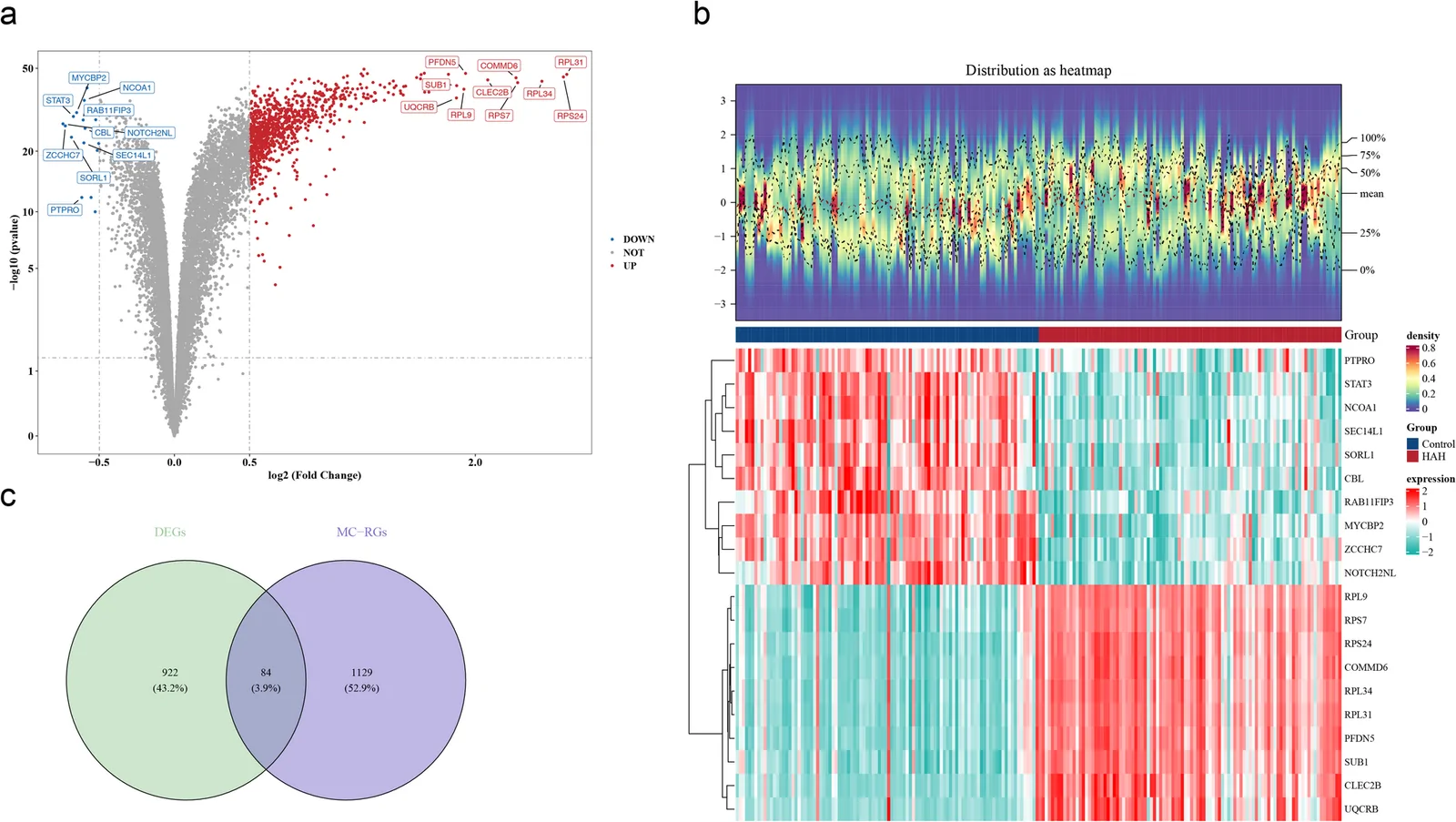
Results of differential expression gene screening and candidate gene identification. **a** The volcano plot present the distribution of differentially expressed genes between high-altitude hypoxia (HAH) and control samples in the GSE46480 dataset. The top 10 upregulated genes (labeled in red) and top 10 downregulated genes (labeled in green) ranked in descending order of |log_2_FC| are annotated. **b** The heatmap presents the expression profiles of the top 10 upregulated and top 10 downregulated differentially expressed genes screened in Fig. 1a across the samples. **c** The intersection of differentially expressed genes and mitotic catastrophe-related genes

### Functional annotation and PPI network analysis of 84 candidate genes

The 84 candidate genes had obvious associations with 236 GO terms and 36 KEGG pathways (P.adj < 0.05). These 236 GO terms encompassed 145 BP, 67 CC and MF terms. The top five BP, CC, and MF terms were ranked separately from smallest to largest by P.adj, and the involved terms included mitotic cell cycle phase transition, mitotic spindle, and protein kinase regulator activity (Fig. 2a, Additional file 3). Similarly, when ranked by P.adj-value from smallest to largest, the top eight KEGG pathways included ubiquitin-mediated proteolysis and prion disease (Fig. 2b, Additional file 4). It is likely that the evolution of HAH is greatly aided by these enhanced terms and pathways. Moreover, a PPI network was created. *HSP90AA1*, *RBX1*, *UBE2D1*, *PSMC6*, and *PSMD14* had the highest degree of connectivity in this network (Fig. 2c).

**Fig. 2:**
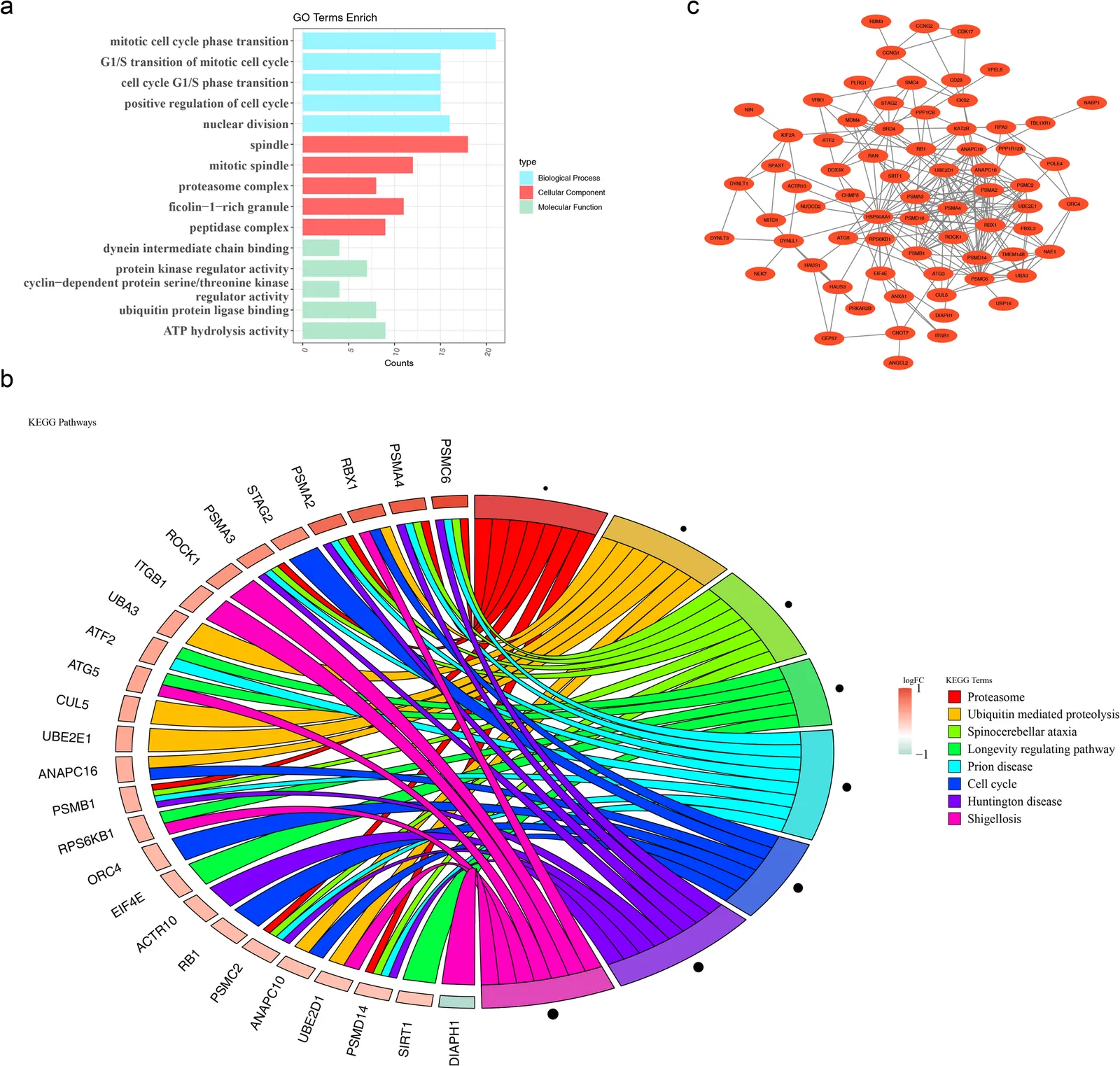
Functional enrichment and protein–protein interaction (PPI) network analysis of candidate genes. **a** GO enrichment analysis bubble plot presenting the top five BP, CC, and MF terms significantly enriched by the 84 candidate genes. The horizontal axis represents the gene ratio, the vertical axis represents the GO terms, the size of the bubble indicates the number of enriched genes, and the shade of color corresponds to P.adj (red indicates greater significance). **b** KEGG pathway enrichment analysis bubble plot presenting the top eight pathways significantly enriched by 84 the candidate genes. The horizontal axis represents the gene ratio, the vertical axis represents the pathway names, the size of the bubble indicates the number of enriched genes, and the shade of color corresponds to P.adj (red indicates greater significance). **c** PPI network presenting the interactions among 84 candidate genes. Nodes represent genes (size is positively correlated with connectivity), edges represent interactions, and the top five genes with the highest connectivity are label

### *DIAPH1* and *MDM4* were identified as biomarkers

In the LASSO regression analysis, seven feature genes 1 (*DIAPH1*, *MDM4*, *UBE2D1*, *RBX1*, *PRKAR2B*, *AIF1*, and *POLE4*) were identified when lambda.min was 0.01911237, and the coefficients of the genes were not penalized to 0 (Fig. 3a–b). Subsequently, 79 feature genes 2 were identified when the accuracy of the SVM-RFE model reached its maximum, and these genes included *RBX1* and *YPEL579* (Fig. 3c). Meanwhile, in the Boruta analysis, 36 feature genes 3 were selected according to the importance ranking of genes, with these genes including *CCNG2* and *TMEM14B* (Fig. 3d). Then, the intersection of feature genes 1, 2, and 3 was taken, and six key genes were obtained: *DIAPH1*, *MDM4*, *RBX1*, *PRKAR2B*, *AIF1*, and *POLE4* (Fig. 3e). In the GSE46480 and GSE103940 datasets, the AUCs exceeded 0.7 for five genes (*DIAPH1*, *MDM4*, *PRKAR2B*, *AIF1*, and *POLE4*), and these genes were considered candidate biomarkers (Fig. 3f–g). The expression of *DIAPH1* and *MDM4* markedly differed between the HAH and control samples in the GSE46480 and GSE103940 datasets, and their expression trends were consistent (*P* < 0.05). Thus, *MDM4* and *DIAPH1* were considered biomarkers (Fig. 3h–i). Notably, *MDM4* and *DIAPH1* expression was higher in control samples.

**Fig. 3:**
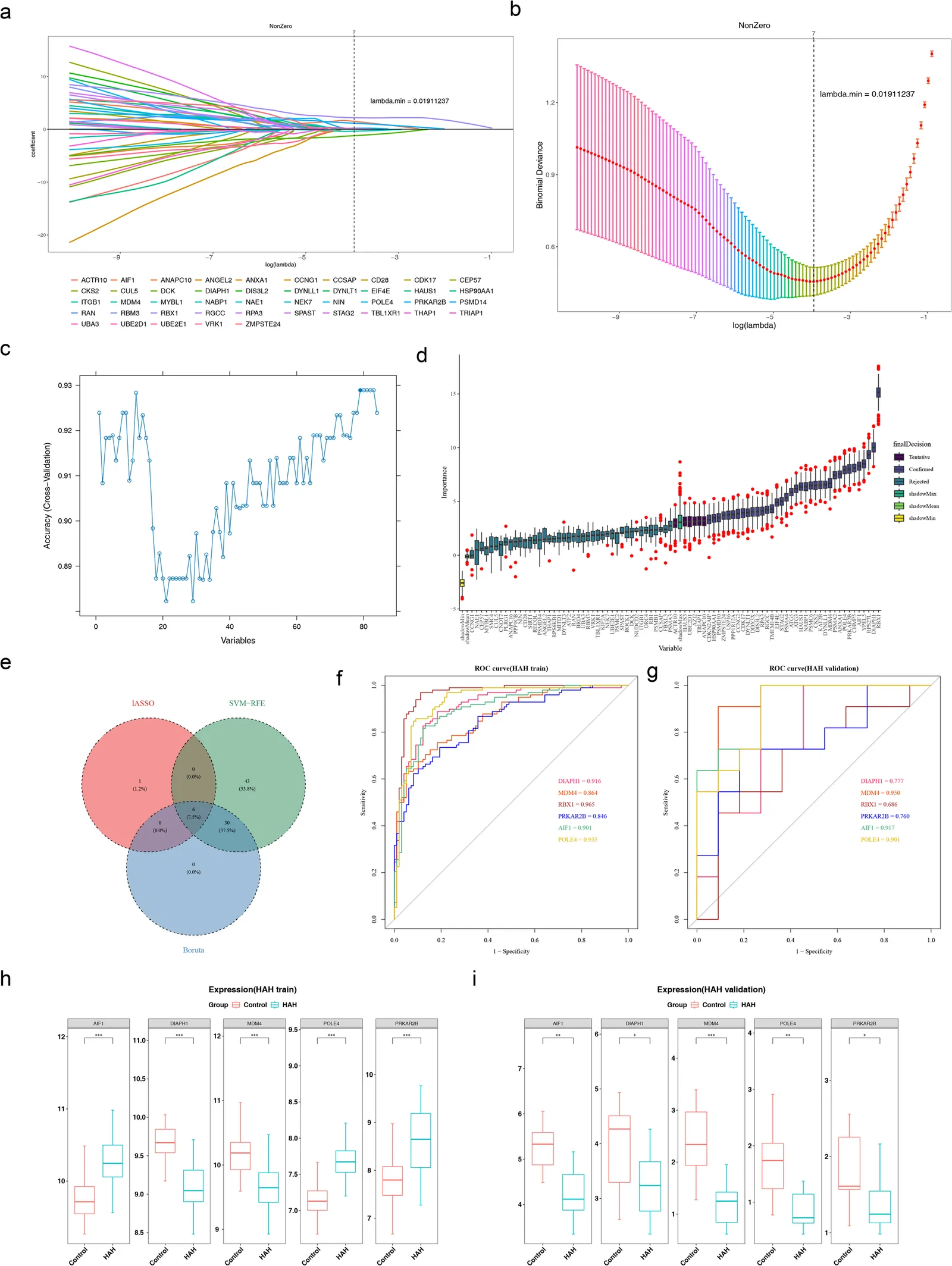
Results of feature gene screening and biomarker identification. **a**–**b** LASSO regression analysis identified seven feature genes 1 when lambda.min = 0.01911237. **c** SVM-RFE revealed 79 feature genes 2 with the highest accuracy. **d** Boruta analysis screened 36 feature genes 3 ranked by importance. **e** Venn diagram illustrating six key genes obtained from the intersection of the three groups of feature genes. **f**–**g** ROC curves of five candidate biomarkers (AUC > 0.7) in the GSE46480 and GSE103940 datasets. **h**–**i** *DIAPH1* and *MDM4* displayed significant expression differences between high-altitude hypoxia (HAH) and control samples in both datasets (*P* < 0.05), and they were finally identified as biomarkers

### The nomogram exhibited excellent predictive capability

A nomogram based on *MDM4* and *DIAPH1* was constructed, as presented in Fig. 4a. In the calibration line, the predicted curve was extremely similar to the ideal curve, as indicated by *P* = 0.23 in the Hosmer–Lemeshow test (Fig. 4b). Regarding the ROC curve, the AUC reached 0.929 (Fig. 4c). The results indicate that the model demonstrates good predictive capability for HAH.

**Fig. 4:**
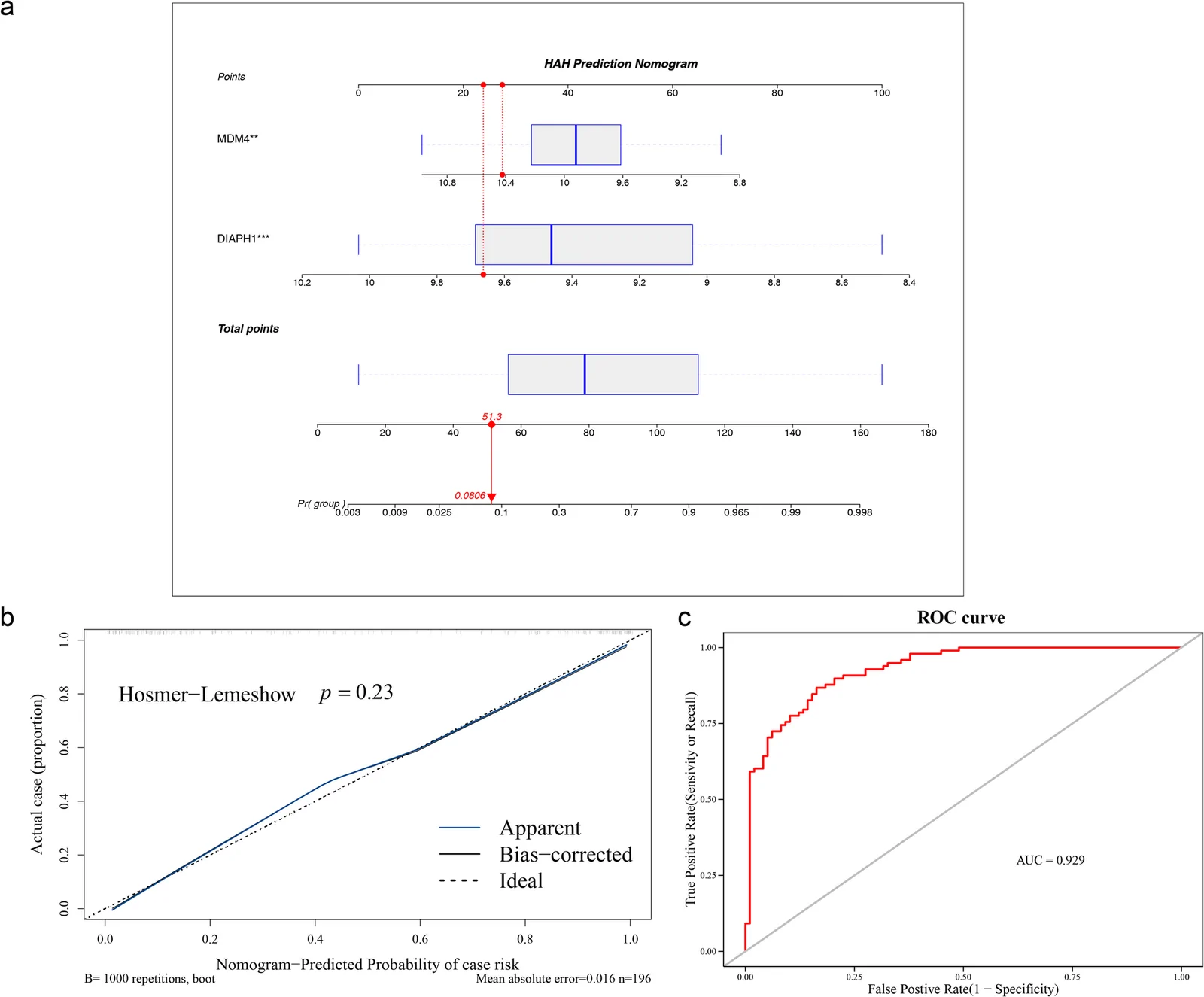
Nomogram for high-altitude hypoxia (HAH) prediction based on *DIAPH1* and *MDM4* and verification of its effectiveness. **a** Nomogram for HAH prediction based on *DIAPH1* and *MDM4*. The nomogram integrates the expression of the two biomarkers, permitting intuitive calculation of the risk of HAH occurrence in individuals. **b** Calibration curve of the nomogram and results of the Hosmer–Lemeshow (HL) test. The predicted curve highly overlaps with the ideal curve (HL test, *P* = 0.23), indicating good consistency between the risk predicted by the model and the actual risk of HAH occurrence. **c** ROC curve of the nomogram. With an AUC of 0.929, the model exhibits strong discriminative ability for HAH occurrence and excellent diagnostic efficacy

### *DIAPH1* and *MDM4* were coenriched in oxidative phosphorylation and other pathways

*DIAPH1* and *MDM4* were enriched in 72 and 49 pathways, respectively (P.adj < 0.05). Subsequently, the pathways were ranked in ascending order of P.adj, and the top five significantly enriched pathways for *DIAPH1* and *MDM4* were identified. The significantly enriched pathways of *DIAPH1* included Parkinson’s disease and oxidative phosphorylation (Fig. 5a, Additional file 5). The significantly enriched pathways of *MDM4* included Parkinson’s disease and phosphatidylinositol signaling system (Fig. 5b, Additional file 6). Additionally, 44 pathways were coenriched for both *DIAPH1* and *MDM4*, including Parkinson’s disease and oxidative phosphorylation. Furthermore, a GGI network was constructed. Genes with functions similar to those of the biomarkers included *MDM2* and *PKD2*, and the functions included regulation of cell cycle arrest and positive regulation of the cell cycle (Fig. 5c). The aforementioned pathways and functions might be pivotal to the development of HAH.

**Fig. 5:**
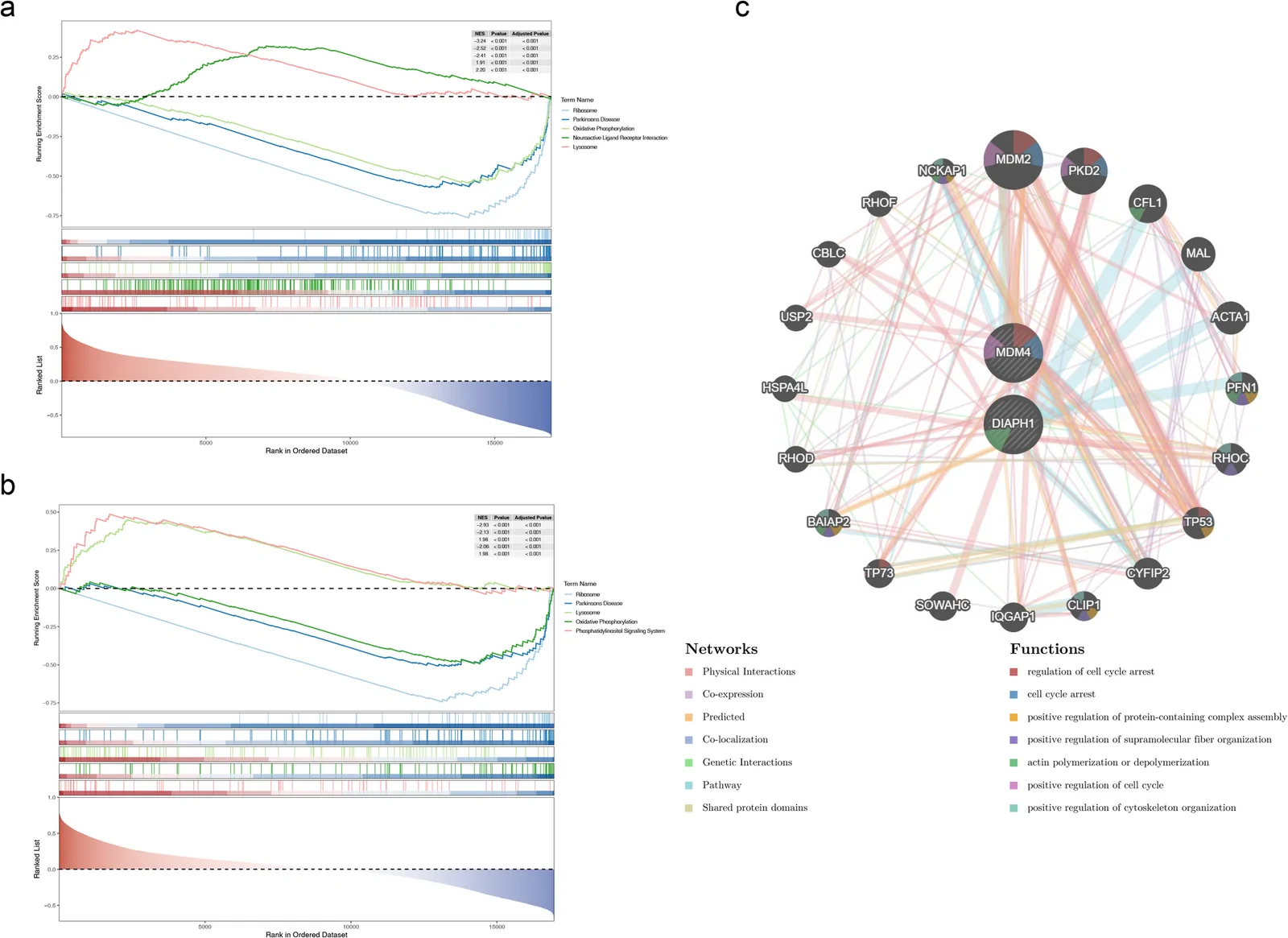
Pathway enrichment and gene–gene interaction (GGI) network analysis of *DIAPH1* and *MDM4*. **a** Top five significantly enriched KEGG pathways for *DIAPH1* ranked by adj.P. **b** Top five significantly enriched KEGG pathways for *MDM4* ranked by adj.P. **c** GGI network displaying genes with functions similar to biomarkers (e.g., *MDM2*, *PKD2*)

### Immune cells correlated with biomarkers

Figure 6a presents the infiltration abundance of 22 immune cell types in HAH and control samples in GSE46480. Eight immune cell types exhibited pronounced differences in infiltration between the HAH and control samples. For instance, the control samples exhibited greater infiltration of monocytes (Fig. 6b). According to Spearman’s correlation analysis, activated memory CD4^+^ T cells and M2 macrophages exhibited the strongest positive correlation (cor = 0.33, *P* < 0.001), whereas resting memory CD4^+^ T cells and neutrophils displayed the strongest negative correlation (cor = − 0.38, *P* < 0.001). Concerning *DIAPH1* and *MDM4*, the strongest positive and negative correlations were observed with resting NK cells (cor > 0.3, *P* < 0.001) and M2 macrophages (cor < − 0.3, *P* < 0.001), respectively (Fig. 6c). In addition, ssGSEA revealed differences in abundance for 22 immune cell types, such as macrophages, mast cells, natural killer cells, and monocytes, between the HAH and control groups (Additional file 7). These findings were consistent with those of CIBERSORT analysis, suggesting the reliability of the immune infiltration analysis. However, these findings were computational predictions, and experimental validation (e.g., flow cytometry) is required in future studies.

**Fig. 6:**
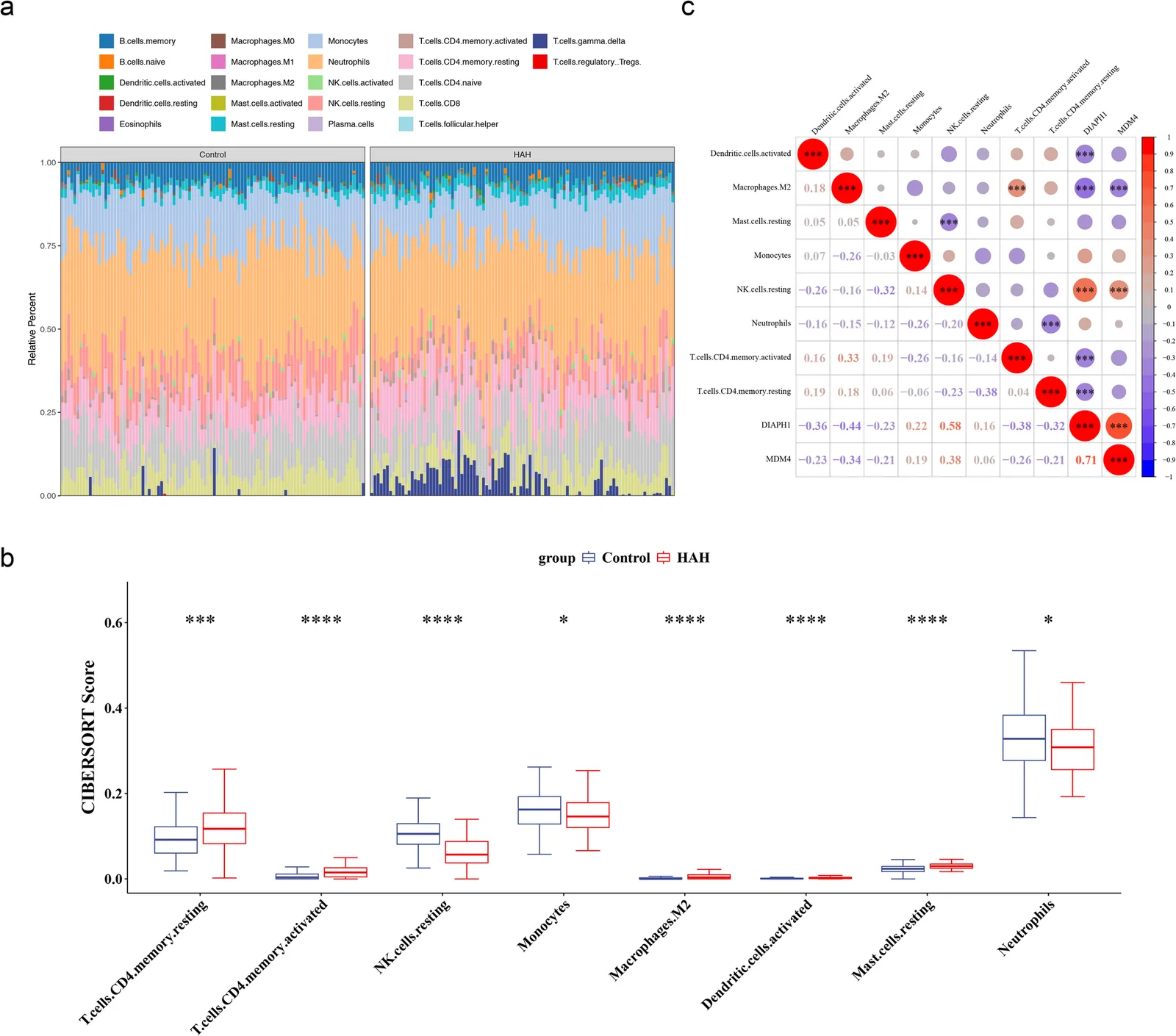
Analysis of immune cell infiltration and its correlation with biomarkers. **a** Infiltration abundance of 22 types of immune cells in high-altitude hypoxia (HAH) and control samples from the GSE46480 dataset. **b** Comparison of the infiltration abundance of eight immune cells between HAH and control samples, *P < 0.05, **P < 0.01, ***P < 0.001, ****P < 0.0001. **c** Spearman correlation analysis between immune cells and biomarkers

### The potential regulatory effects of the biomarkers

The TF–mRNA–miRNA regulatory network included 23 miRNAs, 11 TFs, and 2 mRNAs. In particular, *USF2* and *GATA2* jointly targeted DIAPH1 and MDM4 (Fig. 7a). Next, 2 and 253 drugs were found to target *DIAPH1* and *MDM4*, respectively. Among them, ZINC CTD 00007011 targeted both *DIAPH1* and *MDM4* (Additional file 8). A drug–mRNA network was constructed. The top 15 drugs as indicated by P-value included rescinnamine and lidoflazine (Fig. 7b). The binding energy of molecular docking between rescinnamine and MDM4 was − 9.8 kcal/mol (Fig. 7c), suggesting that the two molecules have strong binding affinity. Moreover, a Chinese herbal medicine–mRNA network was created. Chinese herbal medicines that targeted *DIAPH1* included *Arachniodes exilis* and *Tadehagi triquetrum*, and those that targeted *MDM4* included *Pyrus communis* and *Arctium lappa* (Fig. 7c). However, the aforementioned regulatory networks and drug prediction results were generated by computational analyses, and further experimental validation is required.

**Fig. 7:**
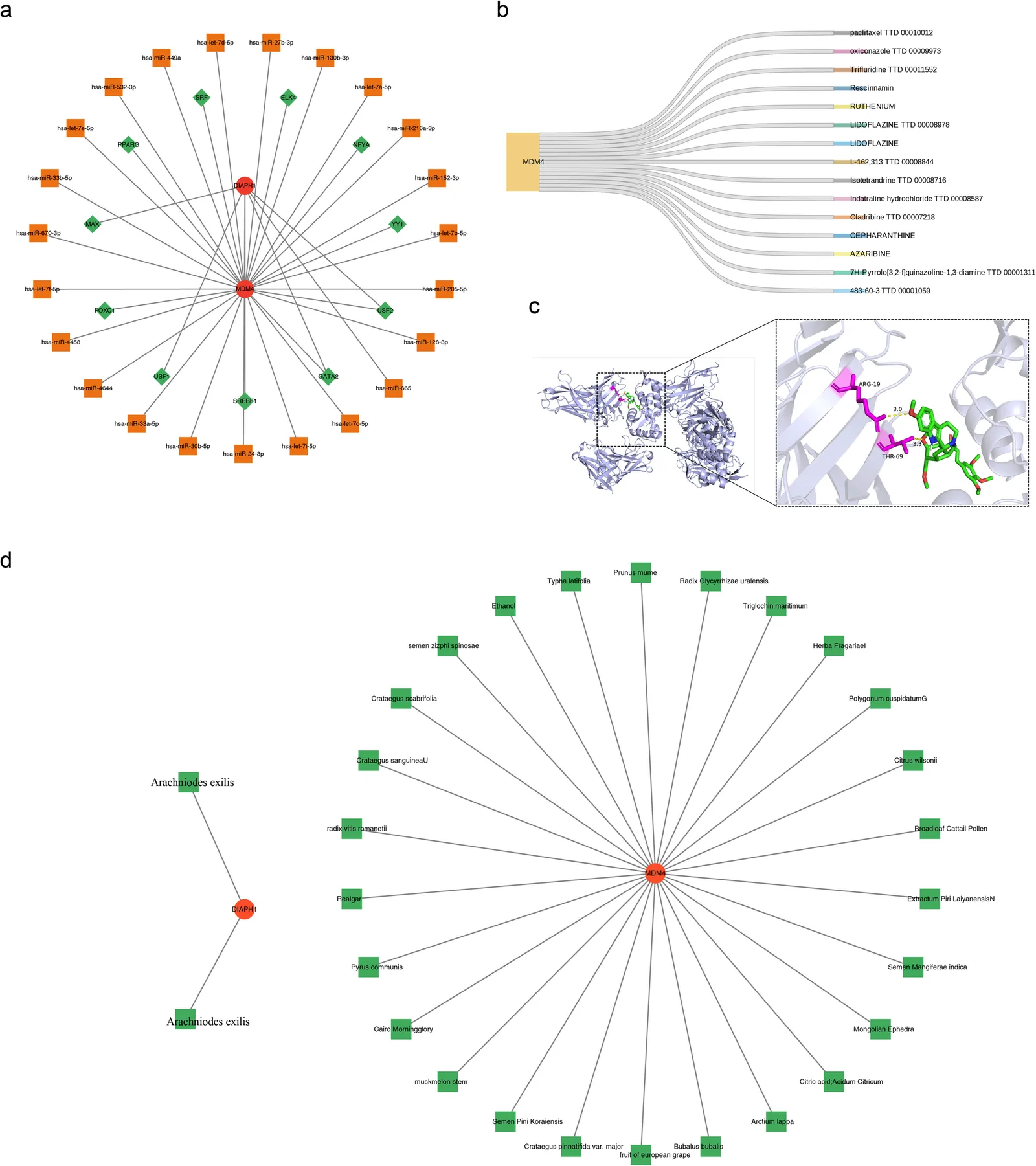
Regulatory network and drug prediction analysis. **a** Transcription factor (TF)–mRNA–miRNA regulatory network presenting interactions among 23 miRNAs, 11 TFs, and 2 mRNAs, with *USF2* and *GATA2* jointly targeting *DIAPH1* and *MDM4*. **b** Drug–mRNA interaction network displaying the top 15 predicted drugs targeting *DIAPH1* and *MDM4*, among which ZINC CTD 00007011 simultaneously targeted both genes. **c** Herb–mRNA interaction network presenting Chinese herbs that target *DIAPH1* and *MDM4*

### Biomarker expression trends were preliminarily validated by RT‑qPCR

The results of RT-qPCR indicated that in clinical samples, the expression of two biomarkers differed significantly between the HAH and control groups (*P* < 0.01). Specifically, *DIAPH1* and *MDM4* were downregulated in the HAH group (Fig. 8). The expression trends of these two genes in clinical samples were consistent with the results obtained in our bioinformatics analysis, supporting the high reliability of the identified through the analysis have high reliability.

**Fig. 8:**
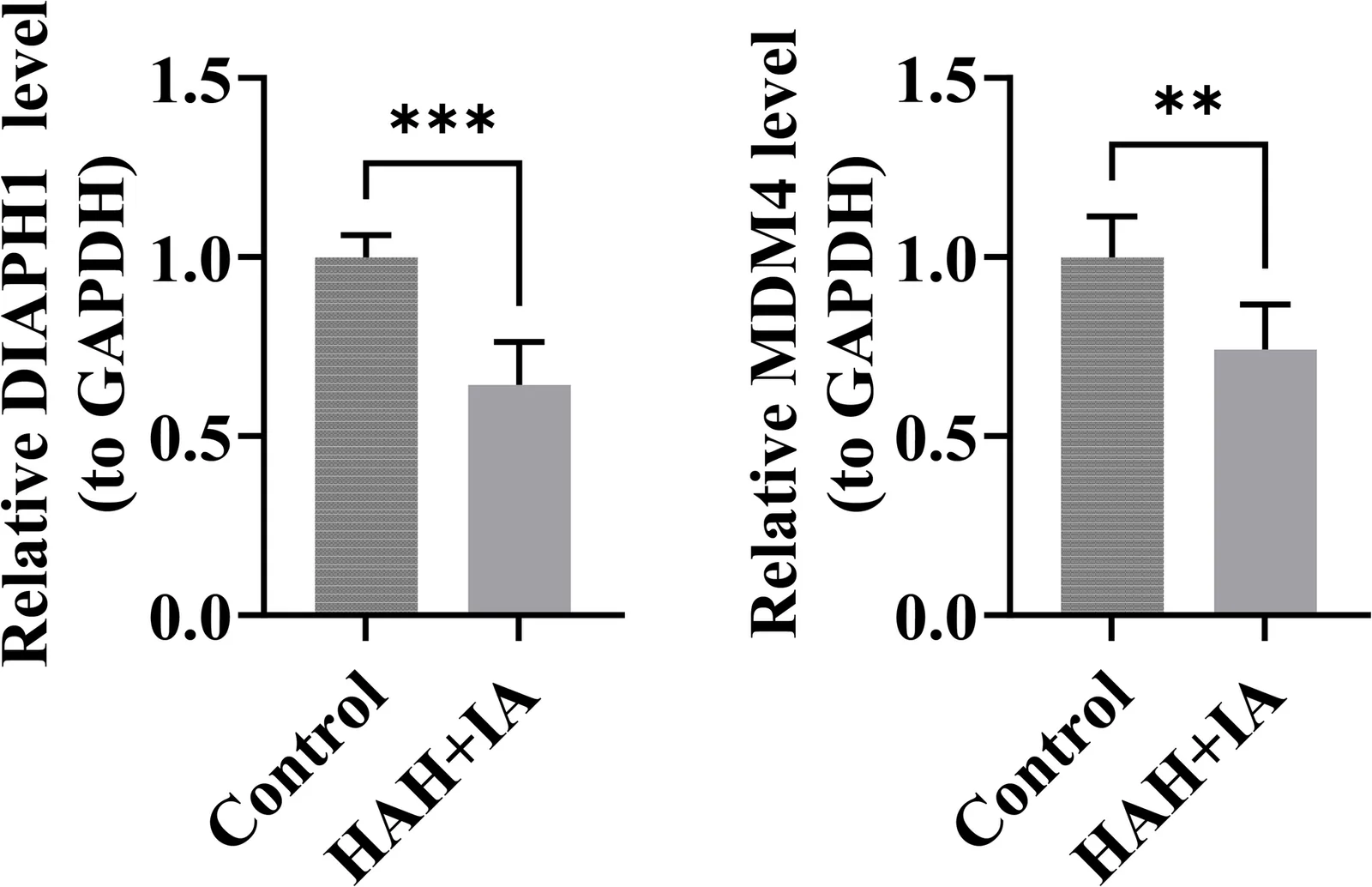
Reverse transcription quantitative polymerase chain reaction validation of gene expression. *P < 0.05, **P < 0.01, ***P < 0.001

## Discussion

HAH is a pathological state caused by a hypoxic environment that significantly increases the risk of diseases, such as chronic mountain sickness [29]. MC is a form of programmed cell death triggered by DNA damage or spindle abnormalities and characterized by multinucleation and genomic instability [30]. Although direct research on their association is limited, hypoxia can indirectly activate MC by inducing DNA damage and cell cycle dysregulation [31], suggesting that MC might be involved in the progression of HAH. This study is the first to identify *DIAPH1* and *MDM4* as potential key MC-related biomarkers in HAH through bioinformatics analysis, and these genes could participate in the occurrence and development of HAH by affecting metabolic, immune, and transcriptional networks, providing new directions for subsequent mechanism research and targeted intervention.

*DIAPH1*, a member of the Formin family, has traditionally been considered to primarily participate in actin polymerization and cytoskeletal reorganization during cell division [32]. However, recent studies have revealed novel functional dimensions of *DIAPH1* under hypoxic stress. Using human induced pluripotent stem cell-derived cardiomyocytes, Yepuri et al. demonstrated that *DIAPH1* directly interacts with the mitochondrial fusion protein MFN2 to regulate the mito-sarcoplasmic/endoplasmic reticulum (Mito-SR/ER) distance [33]. Under hypoxia/reoxygenation conditions, *DIAPH1* silencing significantly increased the Mito-SR/ER distance (from 17.77 to 33.22 nm), attenuated calcium overload and oxidative stress, and improved mitochondrial membrane potential [33]. This finding suggests that *DIAPH1* downregulation could constitute a protective adaptive mechanism in HAH by weakening mitochondrial–ER coupling, reducing mPTP opening, and limiting cell death. Furthermore, the role of the RAGE/DIAPH1 signaling axis in hypoxic injury has gained increasing attention. Ramasamy et al. confirmed that hypoxia-induced advanced glycation end products activate the RAGE–DIAPH1 pathway and upregulate the pro-inflammatory TF Egr1, a process significantly inhibited by *DIAPH1* deficiency [34]. In vascular cells, *DIAPH1* functions as an effector molecule of Rho GTPases, regulating endothelial cell migration and vascular permeability [35]. The present study observed significant downregulation of *DIAPH1* in HAH. Considering its multifaceted roles in mitochondrial quality control, oxidative phosphorylation, and vascular barrier function, we propose that *DIAPH1* might participate in the pathophysiology of HAH through a triple mechanism involving “mitochondria–cytoskeleton–vascular permeability.” This provides a new molecular entry point for understanding HAH-induced vascular leakage and energy metabolism disorders.

As a key negative regulator of p53, *MDM4* fine-tunes cell fate under physiological and stress conditions by directly binding to the p53 protein and inhibiting its transcriptional activation function [36]. Recent studies have revealed that *MDM4* might undergo complex alternative splicing regulation under hypoxic stress, generating protein isoforms with distinct functions. Under hypoxic conditions, *MDM4* exon 6 skipping is increased, generating a short isoform that lacks the p53-binding domain. This isoform both loses its inhibitory effect on p53 and possibly participates in stress responses through p53-independent pathways [37]. In the context of hypoxia-induced oxidative stress, *MDM4* could exhibit bidirectional functional effects. On the one hand, *MDM4* downregulation might relieve the inhibition of p53, activating p53-mediated cell cycle arrest and pro-apoptotic pathways, thereby exacerbating tissue injury. On the other hand, as reviewed by Beyfuss and Hood, p53 can exert tissue-protective effects under hypoxic conditions by transcriptionally activating antioxidant defense genes and cell cycle checkpoint proteins [38]. The downregulation of *MDM4* observed in HAH in the present study could reflect hypoxic regulation of *MDM4* splicing patterns, resulting in a reduction of functional full-length MDM4, which in turn influences cell fate through both p53-dependent and p53-independent pathways. Therefore, *MDM4* might represent a core node linking “hypoxia–p53 regulation–cell cycle checkpoints–MC.” Its expression changes in HAH could provide a novel theoretical framework for understanding the switch in cell death modes induced by HAH.

The results of GSEA indicated that *DIAPH1* and *MDM4* were coenriched in pathways such as oxidative phosphorylation and ribosome, further supporting their important roles in HAH. Oxidative phosphorylation is a biological process that occurs on the inner mitochondrial membrane of aerobic cells, in which the chemical energy released by nutrient oxidation is converted into ATP through the synergistic action of the electron transport chain and ATP synthase. As its core function, oxidative phosphorylation serves as the primary energy source for eukaryotic cells and a central hub of cellular energy metabolism [39]. Studies have demonstrated that the oxidative phosphorylation pathway is associated with HAH. Under hypoxic conditions, the rate of protein synthesis required for various biochemical reactions is significantly slowed and impaired because of limited ATP availability, which is a key basis for tissue damage in HAH [40]. This study further confirmed the key role of oxidative phosphorylation in HAH. Combined with the potential mechanism by which *DIAPH1* downregulation might disrupt mitochondrial structural stability and inhibit oxidative phosphorylation, as well as the role of *MDM4* in regulating mitochondrial function *via* p53, it is hypothesized that abnormal suppression of this pathway could contribute to HAH development by impairing cellular energy supply. This aligns with existing finding linking oxidative phosphorylation to HAH and provides new insights into the mechanism of energy metabolism disorders in HAH.

The ribosome is a cellular organelle responsible for protein synthesis, and it is composed of ribosomal RNA and proteins. Ribosomes translate genetic information from mRNA into specific proteins. As the core sites of protein synthesis, ribosomes can induce stress responses in the hypobaric and hypoxic environment of HAH, in which cells need to synthesize a large number of stress proteins to mitigate hypoxic challenges [41]. The coenrichment of this pathway in this study suggests that it is involved in cellular adaptation or damage in HAH by regulating the synthesis of relevant stress proteins. This finding is consistent with existing conclusions that ribosomes participate in hypoxic stress responses [42], further confirming the potential role of the ribosome pathway in HAH.

Immune infiltration analysis revealed eight types of differential immune cells between HAH and control samples. Resting CD4^+^ memory T cells can be rapidly activated and proliferated upon re-exposure to hypoxic environments, enhancing defenses against repeated hypoxia [39]. Neutrophils are responsible for acute inflammation and damage clearance. Resting CD4^+^ memory T cells and neutrophils exhibit a strong negative correlation, reflecting their functional balance in HAH immune regulation. In HAH, hypoxia can rapidly activate neutrophils through hypoxia-inducible factor and Toll-like receptor [40], causing them to release ROS and proteases to damage tissue barriers and aggravate edema [41].

Acute inflammation consumes T cell pool resources, prompting memory T cells to differentiate into effector phenotypes, and hypoxia exacerbates their exhaustion by inhibiting T cell survival pathways, leading to reductions in resting subsets and weakened immune homeostasis repair capacity [42]. In addition, this imbalance might indirectly promote MC by inducing cell cycle arrest, activating the p53 pathway, or disrupting the spindle checkpoint [43]. M2 macrophages promote repair by secreting factors such as TGF-β and VEGF [44, 45]. Their significant negative correlation with *MDM4* expression, combined with the negative regulation of p53 by *MDM4*, implies that *MDM4* could affect M2 macrophage recruitment and function by influencing the p53 pathway to regulate the release of inflammatory factors, participating in the pathological balance regulation of HAH. These results offer new clues for exploring the regulatory mechanism of immune repair in HAH.

To explore the upstream regulators of the biomarkers and their interactions, we constructed a TF–mRNA–miRNA network. The results demonstrated that the TFs *USF2* and *GATA2* jointly target both *DIAPH1* and *MDM4*. *USF2* regulates cellular metabolism, oxidative stress, and mitochondrial function [46], and its abnormalities could lead to mitochondrial damage and affect cell mitosis [47]. Given the energy metabolism disorders and enhanced oxidative stress in HAH, *USF2* can potentially affect mitochondrial function, oxidative phosphorylation efficiency, and cytoskeletal dynamic balance by precisely regulating *DIAPH1* and *MDM4* expression. *GATA2* plays a key role in regulating hematopoietic function, angiogenesis, and immune homeostasis, and its dysfunction might damage vascular integrity and exacerbate abnormal cell division in HAH [48]. Their synergistic effect could deeply participate in the pathological process of HAH by regulating *DIAPH1* (encoding a key cytoskeletal protein) and *MDM4* (encoding a protein closely related to cell cycle and apoptosis), influencing the occurrence and development of cellular MC.

Among the differential miRNAs, hsa-miR-27b-3p regulates vascular function [49], hsa-miR-532-3p maintains vascular endothelial barrier function [50], hsa-miR-205-5p protects epithelial/endothelial cell homeostasis [50], hsa-miR-30b-5p regulates autophagy and mitochondrial function recovery [51], and hsa-let-7a-5p mediates the core hypoxic inflammatory network [52]. These miRNAs can potentially affect the HAH progression by regulating *DIAPH1* and *MDM4 expression*. Drug and traditional Chinese medicine predictions provide clues for targeted therapy of HAH. Lidoflazine, a calcium channel blocker, can enhance vasodilation, inhibit cellular calcium overload, reduce vascular spasm and tissue damage, and may regulate vascular function by targeting biomarkers to alleviate HAH symptoms [53, 54]. Drugs such as ZINC CTD 00007011 and rescinnamine are also predicted as potential targeted drugs, which might participate in HAH regulation by affecting biomarker function. Traditional Chinese medicine such as *Dryopteris crassirhizoma* and *Parthenocissus semicordata* have been predicted to be associated with the regulation of *DIAPH1*, whereas *Malus pumila* and *Cirsium japonicum* are related to *MDM4* regulation. These traditional Chinese medicines might affect the expression or function of corresponding biomarkers through their active ingredients, providing ideas for the treatment of HAH.

The unique value of this study lies in its pioneering introduction of the MC paradigm into the field of HAH research, moving beyond the traditional focus on apoptosis, necrosis, and pyroptosis. Existing HAH studies have primarily concentrated on damage mechanisms such as oxidative stress, inflammatory responses, and mitochondrial dysfunction [55]. Conversely, this study integrated MC-related gene sets with DEGs in HAH to identify two dual-function biomarkers, *DIAPH1* and *MDM4*, which might simultaneously participate in cytoskeletal dynamics, mitochondria–endoplasmic reticulum coupling, cell cycle checkpoints, and MC regulation, thereby constructing an integrated pathological network of “hypoxia–cytoskeleton–mitochondria–MC.” This study provides a novel perspective for future research: during acute high-altitude exposure, rapidly proliferating hematopoietic and vascular endothelial cells might undergo MC because of hypoxia-induced spindle abnormalities and DNA damage. This both explains the pathological basis of BBB disruption and vascular leakage in HAH and provides a theoretical foundation for developing MC-targeted intervention strategies, such as spindle checkpoint modulators.

However, this study had several limitations. First, although the bioinformatics analysis suggested an association between MC-RGs and HAH, direct experimental evidence confirming that MC is a key driver in the pathological process of HAH is currently lacking, and the causal relationship requires further elucidation. Second, the sample size for RT-qPCR validation was relatively small, and potential confounding factors, such as genetic background and the rate of altitude ascent, were not fully considered, possibly limiting the representativeness of the results. Additionally, this study only verified the expression changes of biomarkers at the mRNA level, lacking experimental corroboration at the protein level. Future studies should employ methods such as western blotting or immunohistochemistry to supplement this validation. Regarding model construction, although the nomogram model demonstrated good diagnostic performance, its generalizability requires further testing in independent cohorts with larger sample sizes using the same platform. Finally, the drug prediction results were based on computational simulations. The actual in vivo effects, pharmacokinetic properties, and therapeutic value of the candidate drugs require validation through cellular experiments and animal models. To address these shortcomings, subsequent studies will expand the clinical sample size with rigorous control of confounding factors, conduct mechanistic investigations into the causal role of MC in HAH, perform protein-level validation, evaluate the diagnostic utility of the nomogram in independent cohorts, and assess the potential application prospects of the predicted drugs through in vitro and in vivo experiments, thereby providing a more substantial scientific basis for the precise prevention and treatment of HAH.

## Conclusions

This study preliminarily identified *DIAPH1* and *MDM4* as potential MC-related biomarkers in HAH using bioinformatics analysis and initial RT-qPCR validation. These two biomarkers might participate in HAH progression by modulating key metabolic, immune, and transcriptional networks. The coenriched pathways, immune infiltration characteristics, regulatory networks, and drug predictions provide new clues for mechanism research and treatment.

## Supplementary Information

Additional file 1. List of mitotic catastrophe-related genes. Additional file 2. Primer sequences used for reverse transcription quantitative polymerase chain reaction validation. Additional file 3. Complete list of GO enrichment terms for candidate genes. Additional file 4. Complete list of KEGG enrichment pathways for candidate genes. Additional file 5. Complete list of GSEA results for DIAPH1. Additional file 6. Complete list of GSEA results for MDM4. Additional file 7. Analysis of immune infiltration using the ssGSEA algorithm. (a) Heatmap of immune cell infiltration. (b) Box plots of differentially abundant immune cells. *P < 0.05, **P < 0.01, ***P < 0.001, ****P < 0.0001. Additional file 8. Drugs predicted to target the biomarkers.

## Data Availability

The datasets utilized in this study were sourced from the GEO database (https://www.ncbi.nlm.nih.gov/geo/).

## Abbreviations

Abbreviation: Full Term
HAH: High-altitude hypoxia
MC: Mitotic catastrophe
MC-RGs: Mitotic catastrophe-related genes
DEGs: Differentially expressed genes
ROC: Receiver operating characteristic
AUC: Area under the curve
RT-qPCR: Reverse transcription quantitative polymerase chain reaction
BBB: Blood-brain barrier
ROS: Reactive oxygen species
ATP: Adenosine triphosphate
UPR: Unfolded protein response
GEO: Gene Expression Omnibus
PBMC: Peripheral blood mononuclear cell
MCDB: Mitotic Catastrophe Database
GO: Gene Ontology
KEGG: Kyoto Encyclopedia of Genes and Genomes
PPI: Protein-protein interaction
LASSO: Least absolute shrinkage and selection operator
SVM-RFE: Support vector machine-recursive feature elimination
GSEA: Gene set enrichment analysis
GGI: Gene-gene interaction
TF: Transcription factor
miRNA: MicroRNA
JASPAR: Joint Annotation of Protein Affinity Regulating
DGIdb: Drug Gene Interaction Database
HL test: Hosmer–Lemeshow test
NES: Normalized enrichment score
HIF-1α: Hypoxia-inducible factor 1-alpha
TLR4: Toll-like receptor 4
VEGF: Vascular endothelial growth factor
TGF-β: Transforming growth factor-beta
